# “*They all laughed and asked me if I enjoyed having sex with those guys*”: Exploring men’s lived experiences when reporting rape to police in South Africa

**DOI:** 10.1371/journal.pone.0235044

**Published:** 2020-08-21

**Authors:** Siyabulela Eric Mgolozeli, Sinegugu Evidence Duma

**Affiliations:** Discipline of Nursing, School of Nursing & Public Health, College of Health Sciences, University of KwaZulu-Natal, Durban, South Africa; Johns Hopkins University Bloomberg School of Public Health, UNITED STATES

## Abstract

Rape is the most demoralizing type of crime violating human rights worldwide. Research has primarily focused on children and women’s experiences of rape, even though victims include men and little documentation available concerning their experiences of reporting these incidents. The study aimed to investigate men’s experiences when reporting rape to the police. An Interpretative Phenomenological Analysis was used to collect and analyze qualitative data from a purposive sample of eleven men who were rape victims. The findings of the study revealed three superordinate and twenty-two subordinate themes. First, motivation for reporting rape to the police included self-protection from re-victimization, being killed, and prevalent prison cultural practice, seeking justice and answers for rape, moral duty, family support, and encouraging reports of the crime. Second, perceived barriers for reporting rape included fear of stigmatization and ridicule, unknown perpetrators, internalized homophobia, men’s preconceived prejudices, perceived justice system delays, fear of being killed, and protecting their reputation. Third, negative experiences when reporting rape included a long waiting period at the reception before opening a case file and the delayed responses of police investigating the rape scene. Also, there was discouragement from the police, disparaging behavior of police, victim-blaming, lack of communication with the victim about case progress and experiences of police homosexual intolerance. The findings show that most men were motivated to report rape to the police despite the perceived barriers and negative experiences they had with the police. Thus, this provides baseline evidence for strategies to be developed to encourage the reporting of rape. Each police station must provide dedicated personnel for professional and sensitive handling of all rape victims, including men. Furthermore, future studies should be conducted to evaluate rape victims’ satisfaction with the services provided by the police.

## Introduction

The World Health Organization (WHO) and United Nations (UN) define rape as a type of crime that constitutes the violation of human rights [1, 2]. Although this definition differs from country to country, in most countries globally, it is regarded as a form of sexual assault involving the ‘penetration of the vagina or anus with any body part or object, or oral penetration by a sex organ of another person without their consent’ [3]. In South Africa, rape is legally defined as any ‘forced or coerced sexual act that involves penetration to any extent or whatsoever by the: genital organs, mouth, anus of another person; any part of the body of one person or any object or any part of the body of an animal into the genital organs or anus of another person; any penetration by the genital organs of an animal into the mouth of another person without consent’ [4]. Any individual who commits this crime against a person of any gender or age should be reported to the police and face the criminal justice system for violation of human rights [4]. The reporting of rape to police is a crucial step that each victim or caregiver in the case of children, should take to involve the criminal justice system for the punishment of perpetrators of this type of crime [5].

In South Africa, a country known as the rape capital of the world [6, 7], the annual statistics from the South African Police Services (SAPS) on rape cases reveal an increase from 40 035 in 2017 and 2018 to 41 583 cases during 2018 and 2019 [8]. However, it is essential to note that these figures are the total of all rape cases for the general population, including women, men, and children [9]. There is no demarcation about the exact number of cases for men, women, or children who reported rape during that specified period [9]. Experts and activists in the field of sexual violence argue that these figures are just the tip of an iceberg, meaning that many rape cases are not reported in the country [10–16]. Furthermore, literature posits that rape cases where men are primary victims are severely underreported and remain invisible [17–20].

Many factors contribute to rape victims not reporting rape to police, and these include poor treatment of rape victims by police officers [21], personal factors such as failure to recognize rape as a crime [22], distrust of the justice system [21, 23] and negativity towards the conviction process [24]. In South Africa, studies on women’s experiences when reporting or seeking help from police reveal women’s dissatisfaction with the police and their conduct when handling their rape cases [25–27]. Women reported having experienced secondary trauma in the hands of police from being threatened, blamed, receiving no empathy, and being discouraged from opening cases [28–30]. These studies on women's experiences when reporting rape or seeking help from police have helped develop strategies to sensitize police on how to handle women reporting rape in South Africa. These approaches include the provision of the specialist units, known as Family Violence, Child Protection and Sexual Offences Units (FCSUs) with trained police (FCS officers) dedicated to investigating and handling women and children [31–33]. Research on men’s experiences in reporting rape to the police is still lagging in South Africa. Although there are lessons to be learned from the studies on women reporting rape to the police to inform the development of strategies to address male-specific non-reporting behaviors, the voices of men who are rape victims are critical and need to be heard. Therefore, this study sought to address this gap by exploring men’s lived experiences when reporting rape to police. It is envisaged that this study may not benefit rape victims only but may also inform the development of better-friendly approaches in handling of rape victims by the police thus improving rape policing in the country and other parts of the world.

## Methodology

### Study setting and design

The current study was conducted across three post-rape care centers in the City of Tshwane Metropolitan Municipality, in the Gauteng province of South Africa. These post-rape-care centers operate 24 hours per day and offer all post-rape services, including counseling, treatment and care, and assistance in reporting rape to the police for women, children, and men. The City of Tshwane Metropolitan Municipality is one of the biggest metropolitan municipalities’ cities in South Africa, with the largest and most diverse population of about 3.3 million [34]. A qualitative research design utilizing the Interpretative Phenomenological Analysis (IPA) was used to explore men's lived experiences when reporting rape to the police. The IPA is a qualitative research approach committed to the examination of how people make sense of their significant life experiences and explores the experience in its terms [35]. It was deemed appropriate to capture men’s lived experiences of reporting rape to the police and it was beneficial in a sense that it allowed the researcher to “get into the participant's’ world of reporting rape to the police” as described by Eatough and Smith [36].

### Sampling and recruitment

Purposive sampling was used to recruit men who were raped and presented for forensic and medical care at the post-rape-care centers following the incident. Initially, fourteen men were recruited, but only eleven agreed to participate in the study, giving a response rate of 79%. The researchers started working with the participants at the end of their first forensic and medical care sessions to avoid interfering with treatment and forensic evidence collection. To be included in the study, the participant had to be a man older than eighteen years, had presented himself within a week of the rape to the facility, with no significant physical injuries requiring immediate admission to the hospital and voluntarily consented to participate in the study. The facility managers received the information about the study, the researcher’s contact details, and were asked to contact the researcher (first author) when a potential participant arrived in the facility. The researcher was on stand-by and would drive to the facility immediately after receiving notification. On arrival, the researcher first established if the potential participant had received the forensic and medical care to ensure that the study did not interfere with the healthcare services required by the participant in the post-rape-care center. The researcher would then introduce himself to the potential participant, explain the purpose and objectives of the study, and request voluntary participation in the study. After that, both the participant and the researcher agreed on an appointment date and venue for the first session. Participants were given a choice of location to indicate where they would be comfortable and available for an interview, and the researcher’s office was made available as an alternative option. Accordingly, five participants were interviewed at the researcher’s office and six in private consultation rooms at the post-rape care centers. Allowing participants to choose venues for data collection in sexual violence is essential as it reduces the chances of secondary victimization and possible coercion [37, 38].

### Data collection

A pilot study was conducted with the first three participants to test the clarity of the research questions and determine whether the recruitment strategy was feasible or not. There were no modifications required to the research questions and recruitment strategy, and the final analysis included the data from the pilot study. The inclusion of pilot data is a common practice in qualitative research because it does not involve any data contamination, and it was more justifiable in this study because the same research questions and recruitment strategy were used [37, 39]. As in most qualitative studies, data collection and analysis were conducted simultaneously between August and November 2018. This concurrent process assisted in the determination of category saturation, which refers to a point where there is a sense of closure because new data yields redundant information [40]. This occurs when the themes and categories emerging from the text become repetitive, and the inclusion of new participants or further engagement provides no unique themes or interpretations [41, 42]. A semi-structured interview guide was used to conduct in-depth face-to-face individual interviews for the collection of qualitative data. The semi-structured interview guide consisted of the following questions: *Have you reported the case to the police; if yes*, *"Can you please tell me what made you report the rape incident to the police*?*" What was your experience when you reported the rape to the police*? *If you did not report*, “*Can you please tell me what made you not report the rape incident to the police*? Further probing questions were asked during the in-depth interviews when necessary for clarification purposes. Interviews were conducted in English, although participants had a choice of using their mother-tongue languages if they preferred. The interviews were audio-recorded and lasted for about one hour, allowing participants enough time to share their experiences on reporting rape to the police. Each interview was transcribed verbatim within 24 hours, while the information could easily be recalled allowing the researcher to initiate preliminary data analysis and be immersed in the data. Each participant’s transcript was saved in a password protected electronic file, using pseudonyms to ensure anonymity. Access to these files was limited to the researcher and the study supervisor only, and will be kept for five years as stipulated by the institutional policy on the storage of research data.

### Data analysis

Manual data analysis was conducted using the IPA guidelines, as described by Smith et al. [43]. First, each transcript was read and reread in order for the researcher to enter the world of the participants. The significant meaning was written on the left-hand margin of each transcript. Second, descriptive, linguistic, and conceptual comments were made on short pieces of text in the transcript. Third, it was checked from beginning to the end to record emerging themes on the right-hand margin. Fourth, the initial notes were transformed into concise phrases to capture the quality of the information in the text. Fifth, the emerging themes were listed according to their connections, then similar themes were clustered together. Some were possible superordinate concepts. Lastly, data units with similar meanings for all cases together were grouped in categories, and patterns examined across all data sets to formulate themes and subthemes. This process was repeated case by case and across cases as part of our commitment to the IPA principles. Three superordinate themes appeared, which represent the men’s lived experiences when reporting rape to police.

### Ethical considerations

The study was approved by the University of KwaZulu-Natal Biomedical Research Ethics Committee (Ref: BE334/18) and Tshwane District Health Research Committee granted permission. The study adhered to all ethical principles for the good conduct of research with humans outlined by the Declaration of Helsinki [44]. Firstly, written informed consent was obtained from participants who voluntarily participated after they were given the information sheet and verbally informed about the study's objectives, benefits and risks, and the recruitment procedure. Each participant received a duplicate copy of the information sheet and the consent form with the details of the researcher, study supervisor, and research ethics committee for easy referral if they wanted more information or clarity. Participants were made aware that their participation in the study was voluntary, and they were free to withdraw at any time if they were uncomfortable. Participants were reimbursed with an amount of ZAR150 for their traveling expenses and time as supported by other researchers in the field of sexual violence [6, 45, 46]. Participants were reassured that their real names would not be made available to any other party during the study and reporting of the research findings; instead, pseudonyms would ensure anonymity and confidentiality. Due to the nature and sensitivity of the study, the risks of the study were that participants could experience discomfort and psychological or emotional distress when sharing their lived experiences of a traumatic event such as rape. The necessary measures, such as a referral system to psychologists at the post-rape care centers, were established for an easy referral of participants who manifested signs of psychological distress during interviews. On identification of a participant who presented with emotional or psychological distress, the researcher stopped the interview and gave the participant enough time and support. After that, the participant was asked if they were comfortable to continue with the interview or prefer to reschedule. Also, the researcher would refer the participant to a psychologist at the post-rape care center where they initially received care. Accordingly, from the eleven participants in this study, seven manifested with emotional distress and were referred to the psychologists after their interviews.

### Measures to ensure trustworthiness

The trustworthiness and rigor of the findings were ensured by applying credibility, confirmability, dependability, and transferability, as outlined by Lincoln and Guba’s model [47]. An intercoder analyzed and identified codes and compared these with those of the researcher to ensure credibility. There was 80% similarity and agreement in the coding, which is an acceptable level of agreement in qualitative data analysis [48]. An independent audit was done by the second author, who is the supervisor of this study and an experienced qualitative and sexual violence researcher. The founders of IPA strongly recommend using independent audits to ensure the credibility of the findings [35]. Member checking was done with eight participants to confirm the findings, and they all confirmed that the researcher interpreted their experiences correctly, and the other three participants were unavailable. The researcher’s interpretations are supported by the participants’ words or verbatim quotes. A semi-structured interview guide ensured that each participant received the same questions to achieve dependability. Also, it ensured consistency throughout the interview process, and feedback from each interview session with its preliminary analysis output was submitted to the supervisor for review. Lastly, an audit trail kept track of all steps taken to reach decisions during analysis to ensure conformability and transferability.

## Findings

The sample comprised of eleven participants whose ages ranged between eighteen and sixty-five years with the majority from age twenty-one to thirty years (Table 1). Three men identified themselves as gay, while the rest were heterosexual men. Five of the heterosexual men were involved in heterosexual relationships, two were widowed and one was married. Two of homosexual men were single and one reported that he was in a relationship. The majority (91%) of participants were black South Africans while only one was a white South African. This is representative of the demographics of the South African population, where black Africans are in the majority. All participants reported their rape to various police stations and through post-rape care centers. However, only eight opened criminal cases for the sexual offences they suffered. The analyzed data revealed three superordinate themes with related subthemes, as presented in Table 2.

**Table 1:** Demographic characteristics of participants (n = 11).

Demographic characteristic	n (%)
**Age**	
<20 years	2 (18)
21 years- 30 years	6 (55)
31 years- 40 years	1 (09)
> 41 years	2 (18)
**Sexual Orientation**	
Homosexual	3 (27)
Heterosexual	8 (73)
**Marital Status**	
Single	2 (18)
Married	1 (09)
Widowed	2 (18)
In heterosexual relationship	5 (45)
In homosexual relationship	1 (09)
**Race**	
Black South African	10 (91)
White South African	1 (09)
**Reported to the police**	
Yes	11 (100)
No	-
**Opened Criminal Cases**	
Yes	8 (73)
No	3 (27)

**Table 2:** Superordinate themes and subordinate themes.

Superordinate themes	Subordinate themes
**1. Motivators for reporting rape to police**	*1*.*1*. *Self-protection from re-victimization*
	*1*.*2*. *Self-protection from being killed*
	*1*.*3*. *Self-protection from prevalent prison cultural practice*
	*1*.*4*. *Seeking justice*
	*1*.*5*. *Seeking answers for rape experience*
	*1*.*6*. *Moral duty*
	*1*.*7*. *Family support and encouragement to report*
2. Perceived barriers for reporting rape to police	*2*.*1*. *Fear of stigmatization*
	*2*.*2*. *Fear of being ridiculed*
	*2*.*3*. *Unknown perpetrators*
	*2*.*4*. *Internalized homophobia*
	*2*.*5*. *Men’s preconceived prejudices*
	*2*.*6*. *Perceived justice system delays*
	*2*.*7*. *Fear of being killed*
	*2*.*8*. *Protecting their own reputation*
3. Negative experiences when reporting rape to police	*3*.*1*. *Long waiting period at the reception before opening case file*
	*3*.*2*. *Delayed response of police in going to the rape scene*
	*3*.*3*. *Discouragement from police*
	*3*.*4*. *Disparaging behavior of police*
	*3*.*5*. *Victim blaming*
	*3*.*6*. *Lack of communication with the victim about the progress of the case*
	*3*.*7*. *Experiences of police homosexual intolerance*

## Motivators for reporting rape to police

The first superordinate theme, “motivators for reporting rape to police” related to the participants’ reasons for reporting their experiences of rape to the police. The theme had seven subordinate themes as follows: self-protection from re-victimization, being killed, and prevalent prison cultural practice; seeking justice; seeking answers for rape experience; moral duty; family support and encouragement to report.

### Self-protection from rape re-victimization

The subordinate theme “self-protection from re-victimization” emerged from data indicating that the participants reported the rape to the police to protect themselves from being attacked for the second time by the same rapists. The participants reported that they were afraid that if the rapists remained free, they would return to rape them again. For example, Reuben, who was raped by a young man in his neighborhood, who is an ex-convict said that he reported the rape to the police to protect himself from being raped daily by the perpetrator. The participant had this to say:

“*In my mind*, *I also thought that then it means this boy will come to my house every night to make me his wife if I do not report him*. *So that is the main reason why I reported this case to the police*. *I was really scared that he will come in to rape me every day*.”

(Reuben, aged sixty-three years).

Another participant, Floyd, who was gang-raped by six guys, indicated that he reported the rape to the police because he feared being raped again, as follows:

“*I reported the case to the police because I was afraid that those guys would rape me again*.”

(Floyd, aged twenty-seven years).

Terres also shared the same sentiments as follows:

“*I became terrified that if I keep quiet about it*, *then it means he will come to do it again*.”

(Terres, aged twenty-seven years).

### Self-protection from being killed

Self-protection from being killed was evident in the data. Participants indicated that they reported the rape to the police due to fear of being killed by the rapists. The rapists used harmful objects such as knives, hammers and daggers to force the victims to succumb to rape and threaten to kill them if they tell other people about the rape incident. For example, Joel, who was raped by his uncle, reported his uncle used a knife to force him to succumb to rape and threatened to kill him if he made any noise. He reported it to police because his uncle had threatened to kill him, so reporting to police was the only alternative he had to ensure his safety. The participant had this to say:

“*My uncle threatened to kill me with the hammer during rape*. *I was really afraid that he will kill me*, *so that is why I reported this case to police officers*.”

(Joel, aged eighteen years).

Reuben also experienced a similar situation where a large knife was used to threaten him when he tried to fight and call for help. The rapist threatened to kill him if he reported or shared the incident with anyone, but due to fear of being killed, he decided to report the rape to police. This is demonstrated by the following quote:

“*When he raped me*, *he had a very big knife*, *and he used it to threaten to kill me when I made noise or tried to fight him off*. *He even told me that he will kill me if I ever try to tell anyone about what he did to me*… *Eeeh but I couldn’t keep it to myself because I was afraid that he will come to finish me off*… *I felt it’s better to report to the police so that I can be protected because he promised to kill me*.”

(Reuben, aged sixty-three years).

Another participant stated that he was raped by someone known in the community as a serial killer who is frequently in jail. He reported the case to the police to protect himself because he knew the rapist as someone who is not afraid to kill, as evidenced by the following quote:

“*Yes*, *I allowed him to do that*…*to rape me rape me because I was afraid that he would kill me with the dagger he had in his hand*. *I’ve known him for a very long time in my [ekasi] community*. *I know that he is not afraid to kill someone*, *so I reported to protect myself because I won’t allow him to do what he did to me again and I know if I refuse*, *he will kill me*. *I know him*, *he can kill someone and pretend as if nothing has happened*.”

(Adam, aged twenty-three years).

### Self-protection from prevalent practice in prison

Participants who were raped in prison reported their rape cases to police to seek protection from the sexual assault practices prevalent behind bars. Participants stated fear of being raped by other fellow prisoners once the rumor surfaced that they were victims of rape. This is a predominant practice in prison, as revealed in the following extracts:

“*You know when things like this happen*, *and people hear about it in prison*, *everyone will think you are cheap and the next thing everyone will want or try his luck in forcing you into sex*. *That’s the way it is in prison*…. *once they hear that you’ve been raped*, *they all want to rape you*. *I was afraid that*, *because it has happened*, *it could happen again*. *That is why I reported so that police can protect me or maybe they can move me to another cell where I will not be at risk*.”

(Ben, aged thirty-four years).

“*I was afraid that the other guys in prison will hear about it and come to use me like what those guys did to me*. *I have been told that the fellow inmates take advantage of you if they hear that you have been raped by other guys*. *So I had to report this so that I don’t get used by other guys in the prison as this seems to be something that they do there once they hear that so and so raped this guy*… *so it attracts other guys to come to do the same thing to you now and again*.”

(Clark, aged nineteen years).

### Seeking justice

Participants indicated that they reported the incident of rape to police to seek justice for the crime they had experienced in the hands of the rapists. Justice sought included punishment so that they would not rape someone again, as indicated in the following quotes:

“*I opened a case with the police because I wanted those guys to be punished and to face the justice*…. *they need to be punished so that they don’t do this to other people*.”

(Floyd, aged twenty-seven years).

“*What he did to me was very bad*, *so I wanted the justice [law] to play its part because he violated my rights as a person and I want him to be punished for that*, *even if it means him being locked in jail forever*. *He is very dangerous to live with us in the location*.”

(Reuben, aged sixty-three years).

“*I reported the case to the police because I wanted him to be punished for what he did to me*. *I know that law will take its part in teaching him a lesson not to do the same thing [that] to anyone else*.”

(Joel, aged eighteen years).

“*Those guys did me wrong*, *so I wanted them to be charged and face justice for what they did to me* …”

(Ben, aged thirty-four years).

Another participant reported the rape to the police so that the rapist could be punished and taught a lesson to prevent him from committing similar assaults. The participant had this to say:

“*I just wanted the guy to be punished and to learn a lesson that rape is not supposed to be committed to anyone*.”

(Adam, aged twenty-three years).

Participants perceived rape as a serious and wrongful act that should not be committed to anyone and warranted punishment. The participants wanted the rapists punished for the crime they had committed, which shows they trust the justice system to convict and prosecute sexual assault crimes such as rape.

### Seeking answers about the rape experience

In this study, participants indicated that they reported the rape to get the truth from the rapists about the sexual crime which they had committed to them as supported by the following quotes:

“*The first reason I had for reporting to the police*, *was that I wanted the truth from those guys as to why they did that to me because I'm a man like them*, *not a girl*.”

(Floyd, aged twenty-seven years).

“*I wanted to know why he did that thing to me because I am not a girl*, *I am a man like him*. *I had no other way to find out but reporting to police was the only option I had to get answers as to why he did this cruel thing to me*. *Why he raped me*? *I mean he is my uncle*.”

(Joel, aged eighteen years).

“*I wanted him to explain to me why he raped me (crying)*. *I wanted him to tell me why he did this thing to me*, *I want him to explain why because I trusted him as a friend*, *and I knew him for a very long time*.”

(Adam, aged twenty-three years).

Another participant indicated that he reported the rape to the police because he wanted answers from the rapist, who later claimed that he thought the victim was a girl. The participant had this to say:

“*I wanted truth as to why he did this to me because after raping me he made claims that he thought I was a girl whereas he knew that I’m not a girl*, *I’m a gay guy*… *I just wanted him to tell the truth as to why he raped me because when he was busy fucking me*, *he knew that he was not fucking a girl*, *he was fucking a guy*. *So*, *I wanted the truth from this guy as to why he raped me and why he is lying to say he thought I was a girl*?”

(Terres, aged twenty-seven years).

### Moral duty

Participants expressed concerns that failure to report the rape to police would mean that they enjoyed it while they did not like what had happened to them. Because rape is a wrong and immoral act that no one deserves, they felt they had a moral obligation to report it. The following quotes supported this:

“*I had to report to the police to prove that I didn’t like that*…*no one like rape*, *I did not like it*. *I was afraid that if I don’t report people will think I wanted it and to make those guys think I enjoyed that whereas I did not like what they did to me*.”

(Floyd, aged twenty-seven years).

“*I reported this thing to the police because I did not like what he did to me*. *You know this thing feels very wrong*, *and I just could not keep quiet about it because he is my uncle*, *and he was not supposed to do this thing to me*.”

(Joel, aged eighteen years).

“*I was afraid that if I don't report this rape case to police people will think I liked and enjoyed it*, *so I did not like or enjoyed what those guys did to me*, *these guys have done me wrong and I do not like it at all*.”

(Clark, aged nineteen years).

“*I forced myself to report this to police because I did not like what those guys did to me and would not want them to do it to someone else*.”

(Ben, aged thirty-four years).

Another participant reported that the rapists did him wrong; therefore, he wanted them prosecuted for brutalizing him. The participant had this to say:

“*The guys who raped me have done me wrong*, *and I did not like what they did to me*. *I really wanted them to be prosecuted for brutalizing me*.”

(Lesley, aged twenty-five years).

### Family support and encouragement to report

Their families supported the participants in this study in reporting their rape to the police. Family members included daughters, sisters, cousins and grandmothers who took the cases into their hands and ensured that the victim reported it to the police when they learned about their rape victimization. This is supported by the following quotes:

“*My daughter told me to open the case at the police station so that the guy can be arrested*. *Although I did not want to*, *my daughter insisted that I report the matter to police officers so that the guy can be arrested*. *Eeeh you know my daughter was very angry and she made sure that I report the case to the police*, *so I had no choice but to report it to the police*.”

(Reuben, aged sixty-three years).

“*My sisters and cousins supported and encouraged me to report the rape to the police because they felt that it’s something*, *I should not keep quiet about*. *So*, *I reported after they persuaded that I lay charges against that guy*. *You know*, *my family is very supportive*, *and they all accompanied me to the police station to report that*, *that guy raped me*.”

(Terres, aged twenty-seven years).

Another participant stated that his family assisted him (aunt and granny) in reporting the rape case to the police after he told them what happened to him:

“*Immediately after I told them at home*, *they were all shocked and felt sorry for me*. *So*, *my aunt and my granny*, *I mean all of them assisted me to report this thing to the police*. *They all agreed that this should be reported to the police*. *Eeeh I reported because of my family* … *They helped me to go to the police station to report*.”

(Joel, aged eighteen years).

## Perceived barriers for reporting rape to police

This second superordinate theme concerned participants’ perceived fears and reasons not to report their rape experiences to the police and lay criminal charges. This superordinate theme yielded eight subordinate themes as follows: fear of stigmatization; fear of being ridiculed; unknown perpetrators internalized homophobia; men’s preconceived prejudices; perceived justice system; fear being killed and protecting own reputation.

### Fear of stigmatization

Participants reported that they were reluctant to report the rape to the police due to fear of being called names and being labeled as useless men as demonstrated in the following quotes:

“*You know people talk a lot*, *so I told myself that I will not go to police to report this thing because I was afraid that everybody will start calling me names*. *I just do not want to be known for this because the moment people get to know this*, *then people will call me different names*.”

(Reuben, aged sixty-three years).

“*I did not want to report this thing to police because I was afraid that they will stigmatize me*. *You know they talk too much*. *So*, *the best thing was not to even go there because I was just scared of them calling me names*.”

(Brown, aged twenty-two years).

Lesley reported that he initially thought the police would call him names or label him as a man who was raped and failed to protect himself as follows:

“*I initially thought they will call me names or label me as that man who was raped*. *I thought they would start calling me as a useless man because I failed to protect myself*.”

(Lesley, aged twenty-five years).

### Fear of being ridiculed

Participants also reported that they did not want to report the rape to the police due to fear of being laughed at, not being taken seriously and had the perception that police would make fun of them as revealed in the following quotes:

“*I couldn’t face police because I was afraid of being laughed by them*. *I know they don’t take people seriously*. *I was just afraid that they will just laugh at me when I tell them that I’ve been raped*.”

(Brown, aged twenty-two years).

“*Since it happened to me*, *I just thought those police will just laugh at me*, *so I was afraid of that because I’ve never seen anyone who have gone through this thing*. *I think those guys (police) will not even try to listen to me or take me seriously*. *I just thought maybe they will laugh at me*. *Who knows*?”

(Reuben, aged sixty-three years).

Another participant mentioned that he was scared that the police would laugh and make fun of him as follows:

“*Those people (police) talk too much*, *so I was afraid that they will talk and laugh about it*. *The only thing in my mind was that they will make fun of me*…*but I was like you know what let me just go and see because I was really not happy with what happened to me*”

(Lesley, aged twenty-five years).

### Unknown perpetrators

The participants expressed concerns about reporting rape to the police related to the fact that they were raped by strangers. This proved to be another barrier as participants stated that they felt their cases would not receive attention because they did not know the perpetrators. For example, Lewis indicated that he did not know the women who raped him, so he felt it would be waste of time to report it to the police because they wouldn’t be able to trace them, as follows:

“*Because I did not know those women*, *I just thought it’s useless to open a case for this thing to police*…*eeh I just do not think police will be able to assist me in tracing those women because I also don’t know them*…*how will they know if I don’t know*? *I think that will make them laugh or blame me for allowing people I didn’t know into my house*…*or maybe they will think I’m the one who followed those women*”

(Lewis, sixty-five years)

Another participant, Lesley, who was gang-raped by three unknown men mentioned that he did not know the guys who raped him and believed this would make it difficult to report. This was a predicament for him because he had no proof and did not know their names. Lesley said that if the names of the perpetrators are unknown or there is little evidence, there is no reason to make a case as follows:

“*It was very difficult because in my mind something was telling me that I couldn’t open or report the case because I do not know those guys*…*it was for the first time to see those faces*, *so I don’t know them*, *I don’t know their names*…*so it’s difficult and I don’t know where will I start to report that to police knowing that I don’t have proof or names of those guys*.”

(Lesley, twenty-five years).

Brown, another participant who was gang-raped by four strangers, also indicated that he did not open a criminal case because he did not know the rapists and felt it was impossible to report rape if the rapist’s identity is unknown. He stated:

“….*so*, *I couldn’t open a case against them because I didn’t know them*. *I don’t think it’s possible to open and report cases like this for someone you don’t know*.”

(Brown, aged twenty-two years).

### Internalized homophobia

This subordinate theme emerged from data in which participants reported that they were reluctant to report the rape to the police because they feared that the police would mistreat them for being gay. The following quotes support this:

“*I was afraid of being mocked and being treated like a useless gay man by those police officers*. *I just thought because I’m gay*, *then they will just say I deserved it*.”

(Phillip, aged twenty-three years).

“*I was very afraid that if I tell them that I’m gay then they will treat me badly and judge me more about this rape and my sexual orientation*.”

(Lesley, twenty-five years).

“*Initially*, *I thought because I’m gay*, *they might not take me seriously or even try to help me*.”

(Terres, aged twenty-seven years).

### Men’s preconceived prejudices

The participants expressed concerns and beliefs that police only protect women and felt that police would not even bother to listen or take them seriously when they report their rape cases to them since they were men. The following quotes confirm this:

“…*remember police always defend women*, *so there is nothing they will do for you as men*, *even if you go to them*, *they will just laugh because the law only protects women*”

(Lewis, sixty-five years)

“*I just thought the police will not even try to listen to me because they protect women when cases like this are reported to them*.”

(Reuben, aged sixty-three years).

“*I just did not think police officers will listen to me when I report that I was raped to them because everyone thinks rape is for women*. *Police always defend women*, *and it is worse in cases like rape*.”

(Lesley, twenty-five years).

Another participant mentioned that reporting rape to police as a man is a waste of time as police do not take male rape cases seriously:

“*Reporting cases like this to police nowadays is a waste of time because they do not take you serious if you are a guy*, *they only take women serious*.”

(Phillip, twenty-three years).

### Perceived justice system delays

The findings of the study revealed concerns and fears that participants had about delays of the criminal justice system with regards to the prosecution of the rapists as per the following quotes:

“*So*, *if I tell police*, *then it means we will be going up and down looking for these women and follow all those long procedures in the court where they will postpone or need witnesses and other things*, *so I’m avoiding all of that*.”

(Lewis, sixty-five years)

“*I did not want to report this thing to police because cases like this take forever*, *and I did not want to frustrate myself*.”

(Reuben, aged sixty-three years)

“*I was thinking that if it comes out of my hands if the police get involved*, *it means that we have to go up and down to courts*. *We must look for the guys*.”

(Brown, aged twenty-two years).

“*I’m still attending school*, *so reporting this thing means going to courts and I’m afraid that the case will take forever*, *and I don’t have that time*.”

(Lesley, aged twenty-five years).

### Fear of being killed

Participants reported that they were scared to report the rape to the police because the rapists threatened to kill them if they reported the rape incident to the police. The rapists used these threats to silence the victims and ensure that they do have the courage to seek help from the police. Some participants knew the rapists, whereas others reported that strangers raped them. However, the threats of killing victims when they report their rape incidents to police could also mean that these rapists knew where these victims lived, putting them further at risk of being endangered by the rapists. For example, the three unknown men who gang-raped Lesley threatened that they would go to his place and kill him if he attempted to report his rape incident to police, as follows:

“*I was afraid that those guys will kill me if I report this thing to police*. *I was very scared because they told me that if they dare hear that I tried to tell the police*, *then they will come to my place and kill me*.”

(Lesley, aged twenty-five years).

Another participant, Phillip, raped by unknown men, also reported that the rapist had threatened to kill him if he attempted to report the rape to police. The participant had this to say:

“*Those guys had knives*, *and they told me that they will kill me if I report this case to the police or try to tell anyone*.”

(Phillip, aged twenty-three years).

Reuben expressed fears about reporting rape to the police because the man who raped him stayed in his neighborhood, and warned him that if he attempted to contact the police, he would kill him, as follows:

“*Eeeeh I did not want to report the incident to the police because the boy stays in the same street as mine and he told me that if I report to the police*, *he will kill me*. *I was very scared that if I call the police*, *he will come straight to my house to kill me*. *You know this boy warned that he will kill me if I report this to police*.”

(Reuben, aged sixty-three years).

Another participant raped by a former friend who is a known serial killer also reported that he was afraid to report the rape to the police because the guy who raped him promised to kill him if he reports the incident. The participant said:

“*I was afraid that he will kill me if I report that he raped me to the police*, *as he initially warned be that if I dare*, *he would finish me off*.”

(Adam, aged twenty-three years).

### Protecting their own reputation

Participants reported that they were reluctant to report their rape to the police because they felt reporting would make other people aware of the rape case and tarnish their reputation. Some participants expressed concerns that reporting it would mean that their communities would know due to police lack of confidentiality. Others mentioned that they had a good personal and professional reputation that they wanted to protect. The following quotes demonstrate this:

“*I did not want people to see me as another person because they would know after reporting this to police*. *I like the way they look at me because it shows they respect me*, *but if they get to know this*, *then it means they will start looking me with another eye*…*these police guys talk too much and they will tell everyone in the location*. *So*, *I thought it’s better not to report so that I protect my reputation and I don’t want people to change the way they look at me*”

(Reuben, aged sixty-three years).

“*I was afraid to report this thing to the police because I thought people would know and then mess up my reputation*. *I’m a well-respected man*, *and my family is well respected in the location*, *and I want it to remain like that*.”

(Lewis, aged sixty-five years).

Lesley was gang-raped by three unknown men and said that he did not want to be the subject of gossip and believed that telling the police about his rape would expose him to other people. Lesley feared that people would then look at him differently, as follows:

“*I just did not want to hear people gossiping about me because they would know after telling police*. *You know if these things get to people*, *eeeeh they start talking all sorts of things about you and this changes the way people look at you*.”

(Lesley, aged twenty-five years).

Another participant, Brown was gang-raped by four unknown men and indicated that he did not regard reporting rape to police as the best move. He believed that it would expose him to other people and was obliged to protect his reputation as a young professional. The participant had this to say:

“*You know I’m a young professional and I have to protect my reputation*, *so I did not think reporting this thing to the police is a wise decision because immediately after reporting this thing to police*, *then many people will know that I was raped by four guys*.”

(Brown, aged twenty-two years).

Participants did not trust the police to keep their rape confidential as they believed that police talk too much and highlighted their fears of gossip from the police being shared with other members of the community.

## Negative experiences with police when reporting rape

This third superordinate theme from data concerned the negative experiences the participants faced when reporting their rape to the police. This superordinate theme yielded seven subordinate themes: long waiting period at the reception before opening case file; the delayed response of the police in going to the rape scene; discouragement from police; disparaging behavior of police; victim-blaming; lack of communication with the victim about the progress of the case and experiences of police homosexual intolerance.

### Long waiting period at the reception when opening a case file

Participants stated that they experienced delays at the police stations when reporting their rape cases. Participants had to wait from one to three hours before police officers could help them open their case files and lay charges. The following quotes support this:

“*I waited for a very long time at the reception until one female officer came in after an hour and she opened the case file*.”

(Floyd, aged twenty-seven years).

“*The first time we entered the police station*, *no one attended to us*. *We just sat there for almost an hour before they could call us to open the case*.”

(Phillip, aged twenty-three years).

Other participants who visited the police station reported that they were told by the police officer to wait on benches where they waited for a long time before the police opened their case files as per the following quotes:

“*When I arrived at the police station reception*, *I was told by another police officer to wait on the benches because they said they were busy*, *yet I could not see what they were busy with*. *I waited for almost an hour until one police officer came from lunch*. *He asked me why was I there*? *I told him that I came to report that I had been raped*. *Eeeh he said I should wait*, *I waited*, *waited*, *waited for another hour before they could help me*.”

(Lesley, aged twenty-five years).

“*The police told us to wait in the benches at the reception*. *I waited there for a very long time*…*something like an hour and half before they could open a file for my case*. *I did not know that it could take so long to report a case like this*.”

(Reuben, aged sixty-three years).

### The delayed response of police to go to the rape scene

Participants also expressed that police delayed accompanying them (rape victims) to the scene of the rape to collect forensic evidence. The delays ranged between three hours and two days. Although some participants were not informed about the reasons for delays, Floyd indicated that the station where he went had only one vehicle which was unfortunately not available at the time. Floyd had this to say:

“*After the female officer assisted me with the opening of the case file*, *she phoned and called his fellow police officers to take us to the place where I was raped to see if they could find any evidence*. *Unfortunately*, *those police officers had left the police station with the only available police vehicle*, *so we had to wait for them until they came very late*. *Eeeh they came after 3 hours and we went to the place where this thing happened*, *but the guys who raped me had long left and there was nothing to find*.”

(Floyd, aged twenty-seven years).

Another participant, Terres, was raped by a guy who is a friend of his friend, reported that he requested police to take him to the rapist’s place where the rape incident occurred. However, the police ignored him until the next shift took over the following day, as follows:

“*As I was waiting*, *I told them that I knew that the guy was going to leave for work or somewhere*. *I requested that we go together to his house because the directions were still clear in my mind*, *and I could still remember the streets*. *They ignored my request the whole night; instead*, *they delayed until the morning*, *the following day when the new shift took over*. *So that shift took me to that guy's house where we found his mother who told us that he had gone to work*.”

(Terres, aged twenty-seven years).

Joel reported that police delayed going to the rape scene for two days. Unfortunately, they could not find the rapist and the flat or scene where the rape occurred could not be accessed because it was locked. The participant had this to say:

“*The police came to my house after two days*, *and they requested us to go to the house where my uncle had raped me*, *but we did not find him*, *and his flat was locked*.”

(Joel, aged eighteen years).

Another participant, Ben, who was raped in prison, described how he was told to wait for the investigating officer during the weekend, who only came to the prison cell on Monday as follows:

“*Those guys raped me on a Saturday night*, *and I was told to wait the whole day on Sunday for the investigating officers*, *but they never came*. *They only came on Monday to the prison cell to ask me to explain how it happened*… *I told them everything*, *and I showed them where the used condoms were*, *they took those as evidence*. *Those guys were moved to another prison cell only after that*. *They could have raped me all over again the whole weekend if they wanted because we were left in the same cell*.”

(Ben, aged thirty-four years).

### Discouragement from police

Participants reported that they experienced discouragement from police when reporting rape. Police were reported to have asked questions that were perceived as disheartening, as evidenced by the following quotes:

“*I immediately went to the police station to report the case*, *and I was hoping that police will assist me in opening the case*, *but they did not open it*, *instead*, *they kept on asking me many questions that discouraged me*.”

(Floyd, aged twenty-seven years).

“*I told them everything that happened*, *but they asked me many useless questions that discouraged me to open the case*. *I could tell from their faces that they were not interested to help me*, *and I felt discouraged to continue with the case because of the questions they were asking me*.”

(Ben, aged thirty-four years).

Another participant, Terres, reported that he felt discouraged to continue with the case as the police kept asking him questions that he perceived to be inappropriate as follows:

“*After my encounter with police*, *I felt so discouraged to even continue with the case or to go to court because they kept on asking me irrelevant questions that discouraged me*.”

(Terres, aged twenty-seven years).

Participants also reported that police told them that their rape cases would take forever, and they felt discouraged to continue or even to open the case as it would take most of their time as follows:

“*They just told me that these cases normally take forever*, *and one must really dedicate time for cases like this to be finalized*.”

(Brown, aged twenty-two years).

“*The police said the case will take forever as we will be going in and out of courts*, *so I got discouraged and lost interest to continue with it*.”

(Adam, aged twenty-three years).

Another participant, Lesley, reported that his interaction with the police made him doubt his story as police did not believe him, as demonstrated by the following quote:

“*When I arrived at the clinic [post-rape-care center]*, *I found two police officers who were assisting some people*. *I spoke to one of them who asked me what happened*, *and I shared everything with him*, *but he was not convinced; instead*, *he said that there is no man who can be raped by women*. *He did not believe me*, *and instead*, *he made me doubt the case even worse*, *and I decided not to open the case or continue talking to them*.”

(Lewis, aged sixty-five years).

### Disparaging behaviors of police

Participants reported experiencing police behavior which was derogatory and belittled the victims as people or trivialized the rape. The following quotes support this:

“*One police officer told me that my case is very weak even if I open it*, *I will not win it*.”

(Floyd, aged twenty-seven years).

“*I was just told by one police officer that my case is very weak*, *and he told me that he cannot waste his energy for a weak case*.”

(Adam, aged twenty-three years).

“*We went to the police station with my sister and the police asked me to explain what happened*, *so I told them everything*, *but they were not recording anything down*. *The police officer told me that my case was very weak*, *more especially because those guys did not penetrate me in the anus*… *so he just said oral penetration will be difficult to prove or to get evidence*.”

(Phillip, twenty-three years).

Other participants also reported that they experienced humiliation where police made jokes and ridiculous comments about their experiences, laughing and making fun of them. The following quotes support this:

“*They all laughed and asked me if I enjoyed having sex with those guys*…*some were even asking me how big their penises were*, *and this made me feel very angry*. *I felt they were making fun of me*.”

(Ben, aged thirty-four years).

“*They were laughing and re-interpreting my story to suit them*. *They were making fun of it as if I was joking that I have been raped*.”

(Terres, aged twenty-seven years).

### Victim-blaming

Participants described their experiences of being blamed by police for the rape they suffered. Participants reported that they were blamed for their association with the perpetrators. This included following the perpetrators to their place or allowing them to get into their (victim’s) place as supported by the following quotes:

“*It's painful what they did to me*, *and unfortunately*, *the police don't see anything wrong; instead*, *they think I wanted to sleep with those guys*. *I told them that I was invited by a friend*, *but they said I knew that those guys are gay*, *and I told them that I did not know*. *So instead of helping me to arrest those guys*, *they started blaming me for going to drink alcohol with a group of gay guys*. *Eeeh now the blame is on me (Crying)* …”

(Floyd, aged twenty-seven years).

“*One thing I did not like*… *the thing that made me not to open the case to police was when they started speculating that I’m the one who followed or invited those women to my house*. *It felt like they were blaming me for what happened to me*.”

(Lewis, aged sixty-five years).

“*They said my story was not making sense and started blaming me that I am the one who followed those guys to smoke weed*.”

(Lesley, aged twenty-five years).

Adam, who was raped by his old friend in prison reported that police, could not believe him when he reported the rape to them, instead they accused him of lying, and they blamed him for the rape incident he had suffered. This is what the participant said:

“*They said nothing like that happens and started pointing fingers on me that I was part of gangsters in the past*…. *they all started to point fingers on me and accusing me of lying*. *I felt very bad because they were all now blaming me for rape whereas the guy who raped me was enjoying his life*.”

(Adam, aged twenty-three years).

### Lack of communication with the victim about the progress of the case

This subordinate theme emerged from data related to the failure of police to update the victims about the progress of their cases. Participants expressed concerns that after they reported their rape cases to the police, they were never given any further information about their cases and whether conviction had taken place or not or involving them during the prosecution process. Some participants indicated that the police had promised to call and inform them about their court date, but never did as demonstrated by the following quotes:

“*They promised me that they will inform me about the court dates*, *but they never came back to me*, *so they are not communicating with me anymore about the progress of the case*…. *Eeeh uhmm I mean no one is updating me about my case*, *I’m just living*.”

(Floyd, aged twenty-seven years)

“*When I reported*, *I was told that I would be called to identify the faces of those guys who raped me*, *and no one has called me*…. *not to even tell me if they found anything*. *It’s very difficult because I didn’t receive any calls updating me to come and identify someone*. *I just receive silence*.”

(Phillip, aged twenty-three years)

Another participant, Clark, who was raped in prison by two fellow prisoners, reported that police never made any follow-up to update him about the way his case would be handled as follows:

“…*they did not make follow-up or even try to communicate with me about the way forward*.”

(Clark, aged nineteen years)

### Police homosexual intolerance

The subordinate theme “police homosexual intolerance” relates to the participants’ experiences with the police. These police demonstrated disapproval and hatred of people who identify as homosexuals, thus showing some severe homophobic elements. This form of hate crime undermines the constitution of the country. For example, Lesley, who was gang-raped by three unknown men and identifies as a gay man, reported he did not tell police officers that he was gay as he heard them passing homophobic comments and mentioning that they hate gay people. He further reported that he decided not to open the rape case due to the homophobic comments made in front of him. The participant had this to say:

“*I did not tell police officers that I'm gay because they were speaking-ill of gay people*, *so I thought they would victimize me since they were passing homophobic comments*. *This made it hard for me to tell them that I am gay because they were making fun about gays*, *and one policeman mentioned that he hates gay people*. *This made me feel very bad*, *and I felt so attacked because I’m also gay*. *Then I was like*, *you know what*? *Let me just leave*, *and I left there without opening the case*.”

(Lesley, aged twenty-five years).

Phillip, another participant who is a gay man, stated that he experienced homophobia from police who asked him inappropriate questions about his sexuality, as demonstrated below:

“*It’s not easy to open cases like this to police because of their bad attitude towards us (gay people)*. *I have always known police as the most homophobic group of people ever*, *and they just gave me that because instead of helping me they started asking me questions why I am gay instead of helping me with the rape case*.”

(Phillip, aged twenty-three years).

Another gay participant, Terres reported that he experienced homophobia from the police and indicated that he would not go back to the police after this negative experience. He said:

“*Those police officers were very homophobic towards me and I do not think I can go back there after what they did to me*.”

(Terres, aged twenty-seven years).

## Discussion

This study aimed to explore men’s lived experiences when reporting rape to police in the City of Tshwane Metropolitan Municipality, South Africa. The findings are encouraging as all participants reported their experiences of rape to the police, which is unusual [49, 50]. However, three of them chose not to open criminal cases for the rape. Some factors such as preconceived fear of stigma, self-protection, men’s preconceived prejudices, anticipated justice system delays, unknown perpetrators and internalized homophobia were barriers for reporting rape to police. These barriers were not only reported by those who chose not open cases, but also by those who had opened cases for other reasons. The negative experiences reported by those who chose to open cases, confirmed these perceived barriers, including long delays, discouragement from police, disparaging behavior of police, victim-blaming, lack of communication with the victim about the case progress and police homosexual intolerance. These barriers and negative experiences could be the reason why some studies have suggested that men are reluctant to report a rape to police or are less likely to report rape than females [51–53].

On a positive note, the findings of this study show that most male rape victims recognize rape as a serious crime that warrants reporting to the police in SA as almost all of them reported their rape to the police. This is encouraging in a country once called the rape capital, where rapists were free to rape without any severe repercussions [7]. In contrast to this study, a survey in the United States of America among male rape victims reported that 76% did not view what had happened to them as a serious crime to be reported to the police or warranted legal proceedings [54]. The reporting of rape by South African male victims could be partly due to the new legal reforms implemented in recent years. An example is the extension of the definition of rape to include the rape of men by the national Criminal Law Act No. 32 of 2007 [55]. This has resulted in the development of interventions, including community awareness campaigns and media statements to educate the public about the new and extended definition of rape in the country. These interventions could be responsible for changing the views of South African men in reporting rape as a crime.

The findings of this study revealed a few South African context-specific factors that motivated male rape victims to report to the police. For instance, reporting for self-protection due to fear of being killed by the rapist is not only peculiar to SA, but it is indicative of the high rates of homicide among men in the country [56, 57]. Fear of being murdered is a reality for most South Africans, and serial rapists and killers are sometimes released early and return to their communities [58]. One participant indicated that the man who raped him was a serial killer who is well known in the community, while another man stated that the young man who raped him had recently returned from jail. South African men, women and children face the dual burden of rape and murder as a crime daily [59–61]. The fear of being killed was also reported as a barrier for not reporting rape to police where participants reported that they feared reporting rape to the police because the rapist had threatened to kill them if they attempt to report the rape to the police. Similarly, the literature reveals that both men and women do not report their rape cases to the police because they fear retaliation or attack by the perpetrator [22, 49, 62]. The criminal justice system in the country must ensure that all rapists are arrested and severely punished, such as serving lifetime imprisonment to ensure the safety of victims. There is little purpose in victims reporting rape to police if the perpetrators face no repercussions and this potentially exposes the victims to homicides and re-victimization. Ensuring lifetime imprisonment of rapists would reduce the prevalence of rape crime and may also assist in reducing the number of murder cases while also encouraging the public to trust the police and criminal justice system of the country.

Most participants stated that they reported their rape to the police to seek justice and for the rapists to be punished or stop them from committing rape again. Such beliefs in the powers of the justice systems to reduce rape crime are noble. For instance, a longitudinal study in the United States of America on reporting crime victimizations to the police and the incidence of future abuse found that police reporting was associated with a 22% reduction in subsequent victimization [63]. Reporting to the police was also more likely to prevent chances of re-victimization as police action could result in the arrest and conviction of the rapist [63]. This highlights the need to strengthen community mobilization approaches tailored to promote reporting rape to the police to curb this crime in the country. Family support was an important motivation for reporting to the police. Most participants said they were encouraged and even accompanied by their family members to report to the police. This is similar to other findings where the disclosure of rape to family and close friends aids in coping during the aftermath of rape [27, 64, 65]. It is reassuring and different from the accounts of other forms of violence against women and children in SA, where family members coerced victims to withdraw or not to report to the police [66–68]. However, some participants indicated that they avoided reporting their rape to the police because they fear losing their respected reputations and family names due to the lack of confidentiality among police. A study by Sable and colleagues found that men had greater confidentiality concerns with regards to reporting rape to the police compared to women [49]. Research in Canada on Women’s Experiences of the Barriers to Reporting Sexual Assault indicated that women feared becoming exposed and wanted their sexual assaults to be kept private [69]. Police handling rape cases should ensure that all reported cases are handled or treated with care and sensitivity while maintaining confidentiality to avoid damaging the reputation and dignity of the victims.

Findings also revealed some perceived barriers held by men concerning reporting their rape to the police. These included fears of stigmatization and ridicule, internalized homophobia, men’s preconceived prejudices, perceived justice system delays, fear of being killed and protecting their reputation. The fear of stigmatization has been reported as one of the most common barriers to reporting of rape to police by male victims. For example, a study in the United States on help‐seeking among male victims of partner abuse found it to be a considerable obstacle that delayed sexually victimized men from seeking support services [70]. Their study found that male rape victims often thought that no one, including the police, viewed them as victims. In this study, participants reported fear of ridicule associated with the fear of being called names or labeled as a man who was raped. For example, one participant reported that: “the best thing was not to even go there because I was just scared of them calling me names”. The literature attests that men avoid reporting rape to the police to avoid social stigma and humiliation, and may even deny the abuse that they have suffered [70–73]. Turchik and Edwards argue that men do not report their rape due to fear of how they may be seen by those close to them, society and police, so they do everything to protect their reputation [73]. There should be measures in place to ensure the privacy of victims and the creation of safe spaces for victims. All rape cases reported must be taken seriously and treated with sensitivity because it takes a brave man to report such experiences, given the social stereotypes associated with male rape [73, 74]. So, men who report a rape to police should be commended and treated with the dignity at all times.

The findings show that victims who were raped by unknown rapists or people they did not know were more less likely to report rape to police. This came out as one of the barriers where participants reported that they could not report and open cases because they did not know people who raped them. This differs with some studies conducted with women where it was reported that women raped by strangers were more likely to report rape to police than women who were raped by acquaintances [22, 75]. Reporting rape to police is a crucial step that victims, families and communities should be taught about because it involves collection of evidence that can be used to trace or locate the offender. One of the reasons why victims are urged to report immediately to the police or rape care center without bathing is to ensure that evidence is not lost as the forensic evidence is dependent on the physical examination of the victim and the collections of samples of bodily fluids that can be used for Deoxyribonucleic Acid (DNA) tests [33, 76, 77]. The police community outreach campaigns should ensure that the public is educated about this important information on rape reporting to ensure that even those who are raped by strangers can get disclosure of who the perpetrators are, depending on their immediate response in reporting.

Internalized homophobia was another barrier to reporting rape to police, where participants feared that the police would mock them for being gay. This could be related to the fact that homophobic violence is more common in South Africa where perpetrators also use rape to show hatred and disapproval of people who belong to the lesbian, gay, bisexual, transgender, queer and intersex (LGBTQI) community [78–80]. A recent study by Mgolozeli and Duma [6] on types of rape experienced by men also found homophobic rape as another type of rape experienced by men in South Africa. Rape is also a form of anti-gay hate crime committed against members of the LGBTQI community, and these victims often choose not to report to the police to avoid disclosing their sexual orientation [81]. The authors argue that gay and lesbian people may fear and be more reluctant to seek help from institutions that were once responsible for policing homosexuality and treat them as criminals that deserved punishment [81]. Sable et al. in their survey study on barriers for reporting rape to the police found high scores linked to the fear of being judged as gay among college students [49]. Although internalized homophobia emerged as a perceived barrier for reporting rape to police, participants also mentioned that they experienced homophobia from the police. This is concerning because police are the members of the state responsible for the protection of the country's citizens and the custodians of law in the country who must respect sexual identities of people, including those of the gay community as stated in the country’s constitution. The fact that these men experience such form of hate from the police reaffirms why gay persons do not report their cases to police. In the literature, it is noted that men who belong to LGBTQI do not seek help from the police because of hostile responses concerning their sexual orientation [81–83]. This calls for more progressive approaches to create safe spaces both in the police and criminal justice system that respects the sexual identities of gay persons. Police should be at the forefront of government initiatives for the promotion of diversity and protection of human rights in the country. The anti-discriminatory policies and laws should be reinforced among the police to ensure that victims are not subjected to elements of discrimination based on their sexual orientation when reporting rape to police.

The findings of this study reveal many challenges experienced by male rape victims when reporting to the police. Previous South African studies on female rape victims on reporting rape to the police also revealed that they encounter difficulties such as dealing with unprofessional police officers, experiencing secondary victimization, including long delays [84, 85]. One participant reported that they had to wait for an extended time for the arrival of another police officer in a police vehicle for transport to the rape scene. Another participant, raped in prison over a weekend, waited for almost two days for an investigating officer to open the case. Such delays are common yet can be avoided if rape and collecting forensic evidence are prioritized [31]. Currently, the country faces shortages of rape kits for collecting forensic evidence. These deficiencies in rape kits and vehicles for transporting police demonstrate the South African Government’s failure to prioritize rape as a crime. However, the same government extended the definition of rape to include men, thus indicating how seriously they consider this crime. The shortage of those very same tools to convict rapists is a mockery of the country’s legal and justice system. In expecting the victims to report the rape to the police as a crime, the failure or delay in collection evidence is equal to secondary victimization of all rape victims in South Africa. The national government should establish a defined budget for the procurement of necessary resources such as rape test kits and vehicles to be strictly used by FCS units to manage rape cases in the police services.

Similar to the findings of this study, the literature attests that rape victims experience policemen’s disparaging behaviors when reporting rape [86–88]. These included discouraging male victims to press charges against the offenders and police officers telling the victims that the cases will take forever. This discouragement from the police, who are custodians of law and safety has adverse effects, including the withdrawal of rape cases, and increases in rape as offenders know that victims do not report, or the cases are withdrawn. The policemen’s disparaging behavior can cause the public to lose faith in the criminal justice system and creates poor cooperation between the citizens and police. The findings of this study also revealed that victims were made to doubt their cases and question whether they will see their day in court. Case retractions and missing police files have been reported in some local urban police stations. For example, a local study found that 33% of rape cases were recorded as undetected, and although documented as having occurred, the police failed to identify the case in two local police stations. As a result, those cases were closed [89]. Although the government instituted the SAPS National Instructions for Sexual Offences as early as 2008 to empower the police on appropriate and sensitive handling of rape victims [90], our findings show that this has not yet been achieved. Participants reported being humiliated and asked repeated judgmental questions by the police who seemed insensitive towards male victims. For example, one participant reported that: “they all laughed and asked me if I enjoyed having sex with those guys” and “some were even asking me how big their penises were”. The participant stated that he felt they were making fun of him. This highlights the humiliation and mockery that can occur when they report their rape experiences to the police, disregarding police conduct rules and regulations as set out in the SAPS National Instructions for Sexual Offences [90]. This type of behavior is reported as one of the barriers to reporting rape to police [49, 91, 92].

Another negative behavior of police is victim-blaming, where police blamed male rape victims and reported in the literature [93–95]. However, most of these studies are quantitative studies with female victims except for one study in the United Kingdom, which found that male rape victims were blamed more than female victims [71]. This victim-blaming behavior was confirmed by one qualitative study identifying victim-blaming statements from the police [71]. Also, male rape victims often blame themselves for their rape experiences [9], so victim-blaming by police can be considered secondary victimization. Therefore, those in management positions in the police must cultivate a culture of responsibility and sensitivity so that rape victims are not subjected to secondary victimization by the police. It may be essential for each police station to have an FCS and victim empowerment officer to ensure that rape victims are attended to by trained officials who can offer sensitive care and understanding of their rape trauma. These dedicated officers may also assist in ensuring that victims are updated about their cases and facilitate follow-ups during the prosecution period. This would also assist in addressing the concerns that participants had about poor communication about the progress of their cases.

## Strengths and limitations

Unlike other studies on this subject, a qualitative research design was specifically chosen using IPA to capture the voices of men regarding their experiences when reporting rape to the police. The use of this approach allowed us to get a deeper understanding about men’s lived experiences when reporting rape to the police, and it assisted in revealing motivational factors, perceived barriers and negative experiences encountered when reporting rape. Although the sample was small (eleven participants) and limited the generalizability of the findings, it is in line with the principles of IPA, which supports the use of small and homogenous samples for in-depth data. The other limitation is in targeting new rape cases followed for one month with no follow-up to obtain information on how their cases were handled during the conviction and prosecution period. Lastly, this study was based on the subjective experiences of eleven participants who had sought help from different police stations in the City of Tshwane Metropolitan Municipality after they experienced rape victimization. The findings from this study may not be generalized to other similar contexts or settings. However, the findings provide a basis for researchers in South Africa and other countries where reporting of rape by men is lagging to conduct similar studies using quantitative methods and bigger sample sizes.

## Conclusion and recommendations

The reporting of rape cases in South Africa will remain a challenge until police personnel and the justice system put measures in place for the handling of all rape victims. The findings of this study provide evidence that men who are rape victims experience secondary victimization when they report the rape to the police and these were further confirmed by their perceived barriers prior to encountering the police or of those who choose not to open criminal cases for the rape they have suffered. This highlights the seriousness of police insensitivity and inappropriate conduct towards rape victims. Consequently, this behavior impacts on the existing problem of the underreporting of rape in the country. This study recommends that community mobilization strategies tailored to promote rape reporting should be established to highlight the plight of rape in the country, including rape victimization of men. These approaches must educate the public about the process of reporting and how forensic evidence is collected and assists in tracing the offenders. Additionally, measures to promote reporting of rape can be drawn from the motivational factors revealed by participants in this study. For example, the themes on deciding to report rape such as self-protection from re-victimization and being killed by perpetrators are powerful factors that should be the aim of campaigns and social debates on reporting of rape to the police. These campaigns should emphasize the importance of reporting rape to police, such as reducing risks for both re-victimization and homicide by the perpetrators. This study further shows that family support has a role in reducing concerns and fears about reporting rape to the police. Participants who disclosed the rape incidents to their families received support, were encouraged to report the cases and even accompanied by relatives to the police stations. Therefore, the campaigns should target the entire community, including families and friends of victims as they have a potential to influence rape reporting behavior.

Police should create conducive and welcoming environments, also known as ‘safe spaces’ that are non-judgmental, less intimidating and less threatening for all rape victims, including men. Such safe spaces may assist in promoting rape reporting behavior in the country not only for men but for all rape victims, including women and children. The authors strongly recommend that police should ensure that every victim who reports a rape to the police is treated with dignity, respect and sensitivity. This also calls for each police station to have at least one trained FCS officer and Victim Empowerment officer who will be able to take statements sensitively and provide support to the victim throughout the process. Regular communication should be in place with each victim regarding the progress of the case. Future studies on male rape victims’ satisfaction with the services received from police should be conducted to inform the development of sensitive care strategies and support frameworks for police dealing with or handling rape victims, including men who are actual or potential rape victims. Furthermore, studies on police attitudes, perceptions and experiences of handling or dealing with rape victims should be conducted in South Africa and in other countries to have baseline data that can be used to inform the development of proper guidelines, interventions and frameworks for policing sexual violence.

## Supporting information

S1 File (DOCX)
