# Supplementary material for: “They all laughed and asked me if I enjoyed having sex with those guys”: Exploring men’s lived experiences when reporting rape to police in South Africa
Source: PLoS One. 2020 Aug 21;15(8):e0235044. doi: 10.1371/journal.pone.0235044 (PMC7444553; doi:10.1371/journal.pone.0235044)
Supplement: S1 File — (DOCX) [file pone.0235044.s001.docx]

**ACKNOWLEDGMENTS**

We would like to thank all the study participants for allowing us into their spaces and sharing their experiences of reporting rape to police with us. We are grateful to the Tshwane Health District for permitting us to conduct this study in their post-rape care centers and the University of KwaZulu-Natal for approving the study and the resources it provided for the successful execution of this study. Our greatest appreciation to Dr Setlola Phoshoko, the District Forensic Medical Services Manager, for ensuring that we could access all the post-rape care facilities. We are indebted to all the facility managers and health professionals in these facilities for being there for us whenever we needed help in the recruitment of participants. Special thanks to Mrs Annah Mabunda, the Assistant Director of Forensic Medical Services at Ekurhuleni Metropolitan Municipality for her efforts in supporting this study, including long late-night calls and emails despite her busy schedule. We are also grateful to Professor Mavis Mulaudzi, Professor Dorricah Peu and Dr Varshika Bhana-Pema for relieving the first author from his teaching responsibilities during the data collection phase and their motivation throughout the study. We would like to acknowledge all members of the IKS and HIV CoP, especially Mabitja Moeta, Mellita Rasweswe, and Miriam Moagi, for their unwavering love, motivation, understanding and emotional support. Lastly, we would like to acknowledge a special friend, Dr Ntlotleng Mabena, who showed interest in the study, for her advice and great insights both as a champion and an advocate for marginalized groups.
